# DBNorm: normalizing high-density oligonucleotide microarray data based on distributions

**DOI:** 10.1186/s12859-017-1912-5

**Published:** 2017-11-29

**Authors:** Qinxue Meng, Daniel Catchpoole, David Skillicorn, Paul J. Kennedy

**Affiliations:** 1 School of Software, Faculty of Engineering and Information Technology and the Centre for Artificial Intelligence, University of Technology Sydney (UTS), PO Box 123, 15 Broadway, Ultimo, NSW 2007 Australia; 2 Children’s Cancer Research Unit, The Children’s Hospital at Westmead, 180 Hawkesbury Rd, Westmead, NSW 2145 Australia; 3 School of Computing, Queen’s University at Kingston, 99 University Ave, ON K7L3N6 Kingston, Canada

**Keywords:** Normalization, Distribution, Gene expression data, R

## Abstract

**Background:**

Data from patients with rare diseases is often produced using different platforms and probe sets because patients are widely distributed in space and time. Aggregating such data requires a method of normalization that makes patient records comparable.

**Results:**

This paper proposed DBNorm, implemented as an R package, is an algorithm that normalizes arbitrarily distributed data to a common, comparable form. Specifically, DBNorm merges data distributions by fitting functions to each of them, and using the probability of each element drawn from the fitted distribution to merge it into a global distribution. DBNorm contains state-of-the-art fitting functions including Polynomial, Fourier and Gaussian distributions, and also allows users to define their own fitting functions if required.

**Conclusions:**

The performance of DBNorm is compared with z-score, average difference, quantile normalization and ComBat on a set of datasets, including several that are publically available. The performance of these normalization methods are compared using statistics, visualization, and classification when class labels are known based on a number of self-generated and public microarray datasets. The experimental results show that DBNorm achieves better normalization results than conventional methods. Finally, the approach has the potential to be applicable outside bioinformatics analysis.

**Electronic supplementary material:**

The online version of this article (doi:10.1186/s12859-017-1912-5) contains supplementary material, which is available to authorized users.

## Background

Personalised or precision medicine aims to find specific therapeutics best-suited for individuals based on their genomic data [10]. It is considered a promising approach for diseases with a genetic component such as cancer. A common approach for quantifiably capturing genomic information is via oligonucleotide microarray technology where gene expression is measured by the signal intensities of probe pairs [12]. Affymetrix GeneChip^1^ microarrays are the most popular and are applied in thousands of bioinformatics studies worldwide.

Although next-generation sequencing and RNA sequencing are increasingly popular, oligonucleotide microarray technology is still in use, and a great deal of microarray data is available. However, it is difficult to compare the results derived from different cohorts (for longitudinal studies) [8], or from multiple sources because of variations in the microarray platforms used or differences in sample preparation or operator sophistication. Because of advances in microarray technology, Affymetrix microarray technologies and probe sets are regularly updated, and it is implausibly expensive to regenerate data after each update. This is especially the case in domains such as rare cancers where data from samples collected infrequently over a long period must be combined to produce enough patient data for comparative analysis.

Normalization is essential in microarray data analysis to enable accurate comparison of expression levels between and within samples [5]. A number of normalization methods have been proposed such as Average Difference (AvgDiff) [c], Total Count (TC) [2], and Trimmed Mean of M values (TMM) [15]. These methods normalize microarray data by aligning the mean and/or variance and work well when the data has the same distribution, but with linear differences in parameters. Spline-based methods [23] were proposed to normalize data by doing regression on local features. However, these methods fail when the distributions themselves differ, which is likely to be the case in practice. Aligning distributions of different shapes is done by defining features of the distributions and then minimizing the differences in these features, for example, using smooth non-linear curves and quantile normalization [17]. Schmid et al. [18] comprehensively compared normalization methods on microarray data.

The performance of such methods depends on good choices of distribution features. They are often inefficient since multiple features must be aligned simultaneously. Most popular software packages for gene expression data analysis and research such as IRON [21], MAAMD [6], AGA [4] and BatchQC [13], use these standard normalization methods. Another widely used normalization software is ComBat [11] which is proposed to standardize mean and variance of microarray data by empirical Bayes. A drawback of this method is that it is hard to control the distribution of data after normalization. A recent survey of Affymetrix microarray data normalization software can be found in [20]. Our approach dealing with distributions opens the way to normalising data generated from different platforms or chip-sets.

We propose an efficient, distribution-based normalization method, DBNorm that works on microarray data from multiple sources regardless of the distributions of the data in each. Because it scales data from different sources into the same distribution they will necessarily also have similar mean, variance and distribution features such as quantiles. We first compare the performance of DBNorm with four state-of-the-art methods: z-score, average difference (AvgDiff) [19], quantile normalization and ComBat on gene expression data derived from diagnostic bone marrow from pediatric Acute Lymphoblastic Leukaemia (ALL) patients on different Affymetrix platforms. As well as the standard approach of statistical evaluation of normalization methods [5, 14], this study also evaluates the performance of normalization methods in the context of downstream utilization of the data for visualization and classification. The performance of DBNorm is also evaluated on a set of benchmark datasets, including a dilution/mixture dataset, a spike-in dataset, and a public acute lymphoblastic leukaemia dataset.

## Implementation

This section describes the main principle of DBNorm, followed by the workflow used to normalize two microarray data samples.

### Distribution-based normalization

Distribution-based normalization transforms values from one scale to another and keeps the order of the magnitudes of the original values unchanged. The probeset and sample value in the dataset that was the minimum before normalisation will remain the minimum after DBNorm. However, the value itself will be different. Similarly, the probeset sample value that was the maximum before normalisation will be the one that is the maximum after DBNorm, albeit taking a different value. In summary, the order of probeset sample values remains the same before and after normalisation. However, the values may differ. Our distribution-based normalization (DBNorm) method is based on this natural principle and is achieved by mapping the probabilities of values in one distribution to another. The fitting functions supported in the DBNorm package are polynomial, Fourier and Gaussian fitting. It also supports fitting distributions using user-defined functions.

The target, desired, distribution might be known in particular contexts, but it is more typical to choose the dataset with the largest number of rows as the best target, transforming the other, smaller datasets to match. DBNorm fits functions to the values of each probeset by regarding them as probability density functions. The values in each column are then scaled to the desired distribution, also regarded as a probability distribution. Consider two columns of microarrays *M*_*1*_ and *M*_*2*_ representing intensities of the same probesets, but with different distributions. Suppose that their fitted probability density functions are *f(∙)* and *g(∙)* respectively. Given an element *m*_*1*_ ∈ *M*_*1*_, the probability of *m*_*1*_ is ${P}_{M_1}\left({m}_1\right)$.

1 $$ {\mathit{\mathsf{P}}}_{{\mathit{\mathsf{M}}}_{\mathsf{1}}}\left({\mathit{\mathsf{m}}}_{\mathsf{1}}\right)={\mathit{\mathsf{P}}}_{{\mathit{\mathsf{M}}}_{\mathsf{1}}}\left[\mathit{\mathsf{t}}\le {\mathit{\mathsf{m}}}_{\mathsf{1}}\right]={\int}_{-\infty}^{{\mathit{\mathsf{m}}}_{\mathsf{1}}}\mathit{\mathsf{f}}\left(\mathit{\mathsf{t}}\right)\mathit{\mathsf{dt}} $$

We want an element *m’*_*1*_ in *M*_*2*_ such that the probability of *m*_*1*_ in *M*_*1*_ is equal to the probability of *m’*_*1*_ in M_2_. This can be expressed in the following equation:

2 $$ {\mathit{\mathsf{P}}}_{{\mathit{\mathsf{M}}}_{\mathsf{1}}}\left({\mathit{\mathsf{m}}}_{\mathsf{1}}\right)={\mathit{\mathsf{P}}}_{{\mathit{\mathsf{M}}}_{\mathsf{2}}}\left({\mathit{\mathsf{m}}}_{\mathsf{1}}^{\prime}\right)=>{\int}_{-\infty}^{{\mathit{\mathsf{m}}}_{\mathsf{1}}}\mathit{\mathsf{f}}\left(\mathit{\mathsf{t}}\right)\mathit{\mathsf{dt}}={\int}_{-\infty}^{{\mathit{\mathsf{m}}}_{\mathsf{1}}^{\prime }}\mathit{\mathsf{g}}\left(\mathit{\mathsf{t}}\right)\mathit{\mathsf{dt}} $$

The microarray column of *M*_*1*_ can be scaled to the same distribution as column *M*_*2*_ via Eq. (2). This transformation necessarily preserves the order of values in each column.

### Workflow of DBNorm

**Fig. 1:**
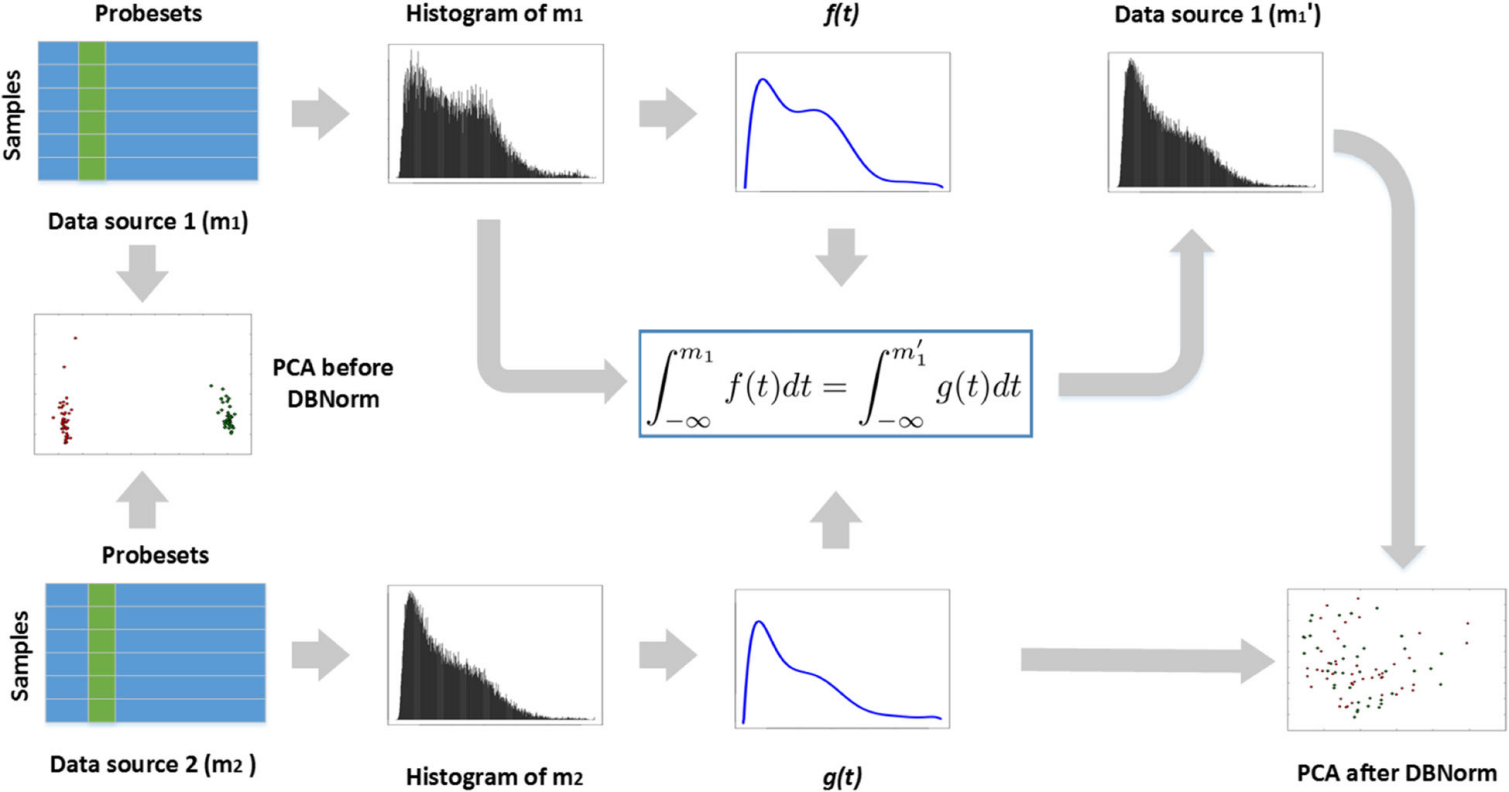
An example application of DBNorm. Probesets from data source 1 (top left) and probesets from data source 2 (bottom left) are visualised with PCA and show platform specific variation. DBNorm takes histograms of probe sets from each data source (second column) and fits a curve to the shape (column 3). The formula (middle column 3) normalises data source 1 to take the shape of data source 2. PCA visualisation of the combined dataset (bottom right) shows reduction in platform specific variation

The DBNorm package was coded to normalize target microarray data by rescaling the column distributions to match a chosen distribution. This process is displayed by an example in Fig. 1.

Suppose that there are two microarray data sources: source 1 (DS1) and source 2 (DS2). Merged together without normalization, they tend to form two clusters with each cluster from one data source. This can be seen in Fig. 1 where high dimensional merged probeset dataset is compressed into two dimensions by Principal Component Analysis (PCA) for visualization.

DBNorm generates distribution information (visible in a histogram). In this example, the distributions DS1 and DS2 are fitted by polynomial functions denoted by *f(t)* and *g(t)*. The element *m*_*1*_ is mapped to *m’*_*1*_ via Eq. (2). The normalized DS1 shows a very similar distribution to DS2. As a result, when we merge normalized DS1 with DS2, the result of a PCA shows that data samples from these two sources mix well, no longer separating based on source.

DBNorm can also rescale microarray data to a standard distribution by choosing *g(t)* to be a standard probability density function, e.g. a normal distribution. Code for this example, and an example of normalizing a data source to a standard distribution are included in the user manual of the DBNorm package.

### Datasets

This section describes the datasets used for evaluating the proposed normalization (DBNorm) method.

The acute lymphoblastic leukaemia (ALL) dataset in this study was collected using Affymetrix microarray data of the diagnostic bone marrow of 146 childhood ALL patients collected by The Children’s Hospital at Westmead, Sydney, Australia^2^ over ten years. As a result, samples were run on several generations of platforms.

Table 1 gives dataset details. In particular, the number of patients whose samples were run on each platform is small. To evaluate the performance of normalization methods based on classification accuracy, we consider relapse as a class label.

**Table 1:** Westmead Acute Lymphoblastic Leukaemia (ALL) dataset

Platform	Patients	Probesets	Relapse (Y/N)
Affymetrix_U133A	18	22,283	6/12
Affymetrix_U133A2	44	22,277	13/31
Affymetrix_U133Plus2	44	54,675	6/38
Affymetrix_HG1ST	40	33,297	8/32

We also use a set of public domain datasets whose details are listed in Table 2. The dilution/mixture dataset is from 75 Affymetrix HG-U95A (version 2) arrays from one source of RNA derived from liver with one microarray (source A) having half the amount of RNA than source B. These two sources are hybridized to human array (HGU95A) covering 12,625 genes with 201,800 Affymetrix microarray probes [7]. Specifically, there are three scanners and each one array replicate was processed in different scanner. The data can be found in Bioconductor.^3^

**Table 2:** Public domain datasets

Public domain datasets	Platform	Samples	Microarray
Dilution/mixture	HG-U95A cRNA data source A	75	201,800
	HG-U95A cRNA data source B	75	201,800
Spike-in	HGU133	42	248,152
	HGU95	59	201,807
Public ALL	HG-U133A	20	22,283
	HG-U133B	20	22,283

The spike-in dataset [9] is generated on Affymetrix platforms HGU133 and HGU95. The platform HGU133 data contains 42 samples with 22,300 genes and 248,152 Affymetrix microarray probes, while the platform HGU95 data contains 59 samples with 12,626 genes and 201,807 Affymetrix microarray probes. Each RNA source has been contaminated with additional RNA resulting in a highly disproportionately mixed set of genes which light up in non-uniform distributions. This dataset can be found on Bioconductor.^4^

The public acute lymphoblastic leukemia (ALL) dataset contains 20 samples of the MLL subtype across two Affymetrix microarray platforms: HG-U133A and HG-U133B. The dataset is published in [16] and can be accessed via Bioconductor^5^ as well. To construct an artificially uniform but widely dispersed dataset to test the normalization, we compared 22,283 random and non-matching probesets between the two platforms.

## Results and discussion

### Applying DBNorm to the Westmead ALL dataset

As different platforms have different probesets (Table 1), only the common probes from different platforms can be used to merge data from them. For Affymetrix microarray data, there are 11,288 common probes for the platforms U133A, U133A2, U133Plus2 and HG1ST.

**Fig. 2:**
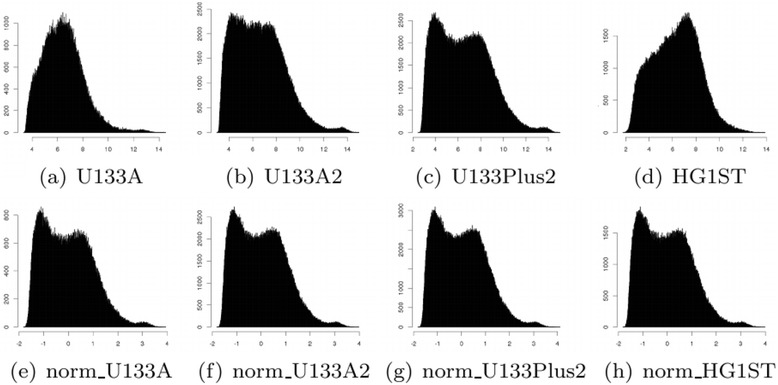
Plots of distributions of all genes across all patients from platform U133A, U133A2, U133Plus2 and HG1ST before and after normalization by the proposed distribution-based normalization method

Table 3 gives the statistics of the common probesets for each platform. It shows that statistics differ across platforms as expected. Platforms U133A2 and U133Plus2 are more similar to one another than U133A, and platform HG1ST is quite different from all three. This is also confirmed in Fig. 2 where the distribution of all samples from U133A2 (Fig. 2(b)) is similar to the distribution of all samples from U133Plus2 (Fig. 2(c)) and the distribution of all samples from HG1ST (Fig. 2(d)) is quite different from the others. Normalization is essential for downstream analysis.

**Table 3:** Statistics of the ALL microarray data

Platform	Probesets	min	max	mean	std
Affymetrix_U133A	11,288	3.335	14.504	6.457	1.606
Affymetrix_U133A2	11,288	2.961	14.946	6.577	2.075
Affymetrix_U133Plus2	11,288	2.451	15.019	6.515	2.294
Affymetrix_HG1ST	11,288	1.749	13.874	6.274	1.966

We choose platform U133Plus2 as the target and transform the other datasets to match (Fig. 2(g)). This shows the ability of DBNorm to match an arbitrary distribution. We assume a polynomial probability density function for all platforms. Figure 2(e), (f) and (h) show the distributions of the datasets from platforms U133A, U133A2 and HG1ST respectively after normalization. It is clear that these three datasets now have a similar distribution to the dataset from platform U133Plus2.

We use the Kullback–Leibler Divergence to calculate the distribution similarities for each platform before and after normalization; the results are given in Table 4. Before normalization, the distributions are different. The biggest difference is between platforms HG1ST and U133Plus2, 0.2300, followed by the difference (0.1475) between platforms U133A and U133Plus2, and the difference (0.1177) between platforms U133A2 and U133Plus2.

**Table 4:** Results of Kullback–Leibler Divergence

Distribution Comparsion	Before normalization	After normalization
U133A vs. U133Plus2	0.1475	0.0001
U133A2 vs. U133Plus2	0.1177	0.0001
HG1ST vs. U133Plus2	0.2300	0.0007

We further compare these standard normalization techniques: z-score, Average Difference (AvgDiff), ComBat and Quantile normalization with DBNorm. First, we compare the statistical properties of the distributions produced by these techniques (Table 5). All normalize data into similar ranges, but there are non-trivial differences in the resulting distributions.

**Table 5:** Statistical evaluation of normalization methods

Method	Min.	1st Qu.	Median	Mean	3rd Qu.	Max.
z-score	−1.741	−0.739	−0.112	0.000	0.553	4.688
AvgDiff	−1.827	−0.742	−0.097	0.000	0.591	4.689
Quantile	−1.628	−0.846	−0.082	0.000	0.673	3.910
ComBat	−2.536	−0.811	−0.064	0.000	0.681	4.910
DBNorm	−1.636	−0.854	−0.073	0.000	0.690	3.748

**Fig. 3:**
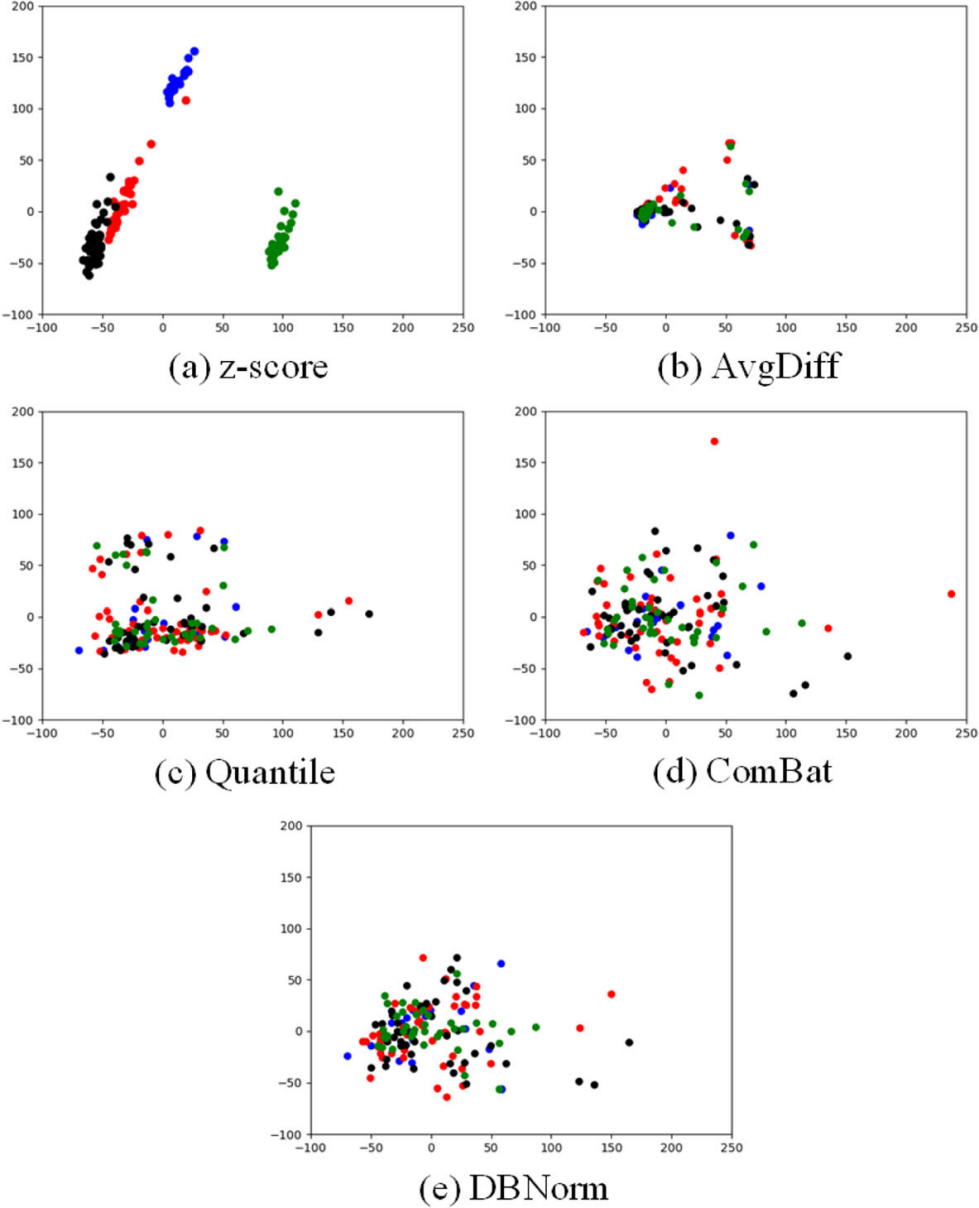
Visualizing gene expression data by principal component analysis (PCA). In the figure, green dots are patient data from HG1ST; blue dots are from U133A; red dots are from U133A2; and black dots are from U133Plus2

Visualization provides another way to assess the quality of normalizations. We compare the results of z-score normalization, AffyDiff, Quantile, ComBat and DBNorm, and visualize them using principal component analysis (PCA) [1] in two dimensions. In Fig. 3, patients whose data was generated from different platforms are marked in different colours. Green dots are patient’s data from HG1ST; blue dots from U133A; red dots from U133A2; and black dots from U133Plus2.

The result of applying z-score normalization only is illustrated in Fig. 3(a) where the integrated data shows marked platform artifacts. In the diagram, there are three major clusters. The cluster at the top-left corner is patient data from HG1ST, and is far from the other two clusters; the distribution of HG1ST is quite different from the other three platforms. The cluster at the bottom represents the patient data from U133A, and the remaining cluster is for patient data from U133A2 and U133Plus2. Although patients from these two platforms are close enough to form one cluster, they are not well mixed. Fig. 3(b) and (c) illustrate the result for AvgDiff and quantile normalization respectively. They disperse the data regardless of the platform to some extent but not much. Figures 3(d) and (e) are the results of ComBat and DBNorm. Both of them can mix patients from different platforms together and DBNorm can compress patents in a smaller area because after normalization DBNorm can achieve a narrow value range (Table 5). Meanwhile, DBNorm provides an explicit way to control which distribution the input data is scaled into while ComBat does not.

Classification can further evaluate the quality of normalizations. Specifically, if data samples from different platforms are not well mixed, classifiers must differentiate the important variation between records of different classes from the irrelevant variation between records in different (platform-based) clusters. We compare prediction accuracy before and after normalization for the Westmead dataset, which is the only dataset for which we have a class label, **relapse**. We use a soft margin Support Vector Machine (SVM) [3] with a Gaussian kernel as the classification technique to classify the normalized dataset based on relapse. To avoid the influence of randomness, we applied Leave-one-out-cross-validation [22] to evaluate the average performance of the built classifier by running 146 times as there are 146 patients. The reason for choosing LOOCV instead of the commonly used K-fold cross validation is that the dataset is somewhat imbalanced, with 33 relapsing patients and 113 patients who did not relapse and LOOCV can make full use of the whole dataset. Also, because of the imbalance issue, the performance of the classifier is evaluated by accuracy, F-measure, ROC AUC (Table 6) and *p*-value with confidence interval as 0.95 (Table 7). Table 6 shows the accuracy, F-measure and AUC averaged over the training set and test set sections of LOOCV respectively for each of the methods. The AUC value for the test portion for a method, for example, is the average of the AUC value for each patient (i.e. each left out point). Table 7 shows the p-value derived from a paired t-test comparing the mean of the sample of AUC values over patients for the test portion for each method compared with DBNorm. It demonstrates that classification after using DBNorm is statistically significantly better than the other methods.

**Table 6:** Evaluating normalization by SVM

Method	Averaged LOOCV Training			Averaged LOOCV Test		
	Accuracy	F-measure	ROC AUC	Accuracy	F-measure	ROC AUC
Unnormalized	0.57	0.31	0.63	0.19	0.08	0.22
z-score	0.79	0.49	0.81	0.23	0.11	0.26
AvgDiff	0.87	0.58	0.90	0.41	0.29	0.45
Quantile	0.91	0.60	0.93	0.79	0.52	0.82
ComBat	0.94	0.67	0.95	0.81	0.61	0.85
DBNorm	0.97	0.72	0.98	0.84	0.73	0.87

**Table 7:** *p*-value of comparing DBNorm with the other normalization methods in terms of ROC AUC on test dataset

	Unnormalized	z-score	AvgDiff	Quantile	ComBat
DBNorm	2.2 × 10^−16^	6.7 × 10^−16^	4.5 × 10^−13^	2.1 × 10^−14^	2.9 × 10^−13^

Table 6 shows that the performance of the classifiers on test dataset differ. DBNorm normalization achieves the highest accuracy (0.84) and F-measure (0.73) followed by ComBat, Quantile normalization, with AvgDiff and z-score perform poorly. The classification result on unnormalized data is worst which shows that normalization is of great necessity in data preprocessing.

### Applying DBNorm on public domain datasets

We now compare the performance of the normalization methods on several public-domain datasets. The performance of these normalized methods is evaluated from statistical level including distributions and MA plot due to lack of class labels. MA plot visualizes the differences between measures taken into two selected samples by transforming the data onto M (log ratio) and A (mean average) scales which is considered as a major way to compare the difference of two gene-expression sources.

#### Dilution/mixture dataset

**Fig. 4:**
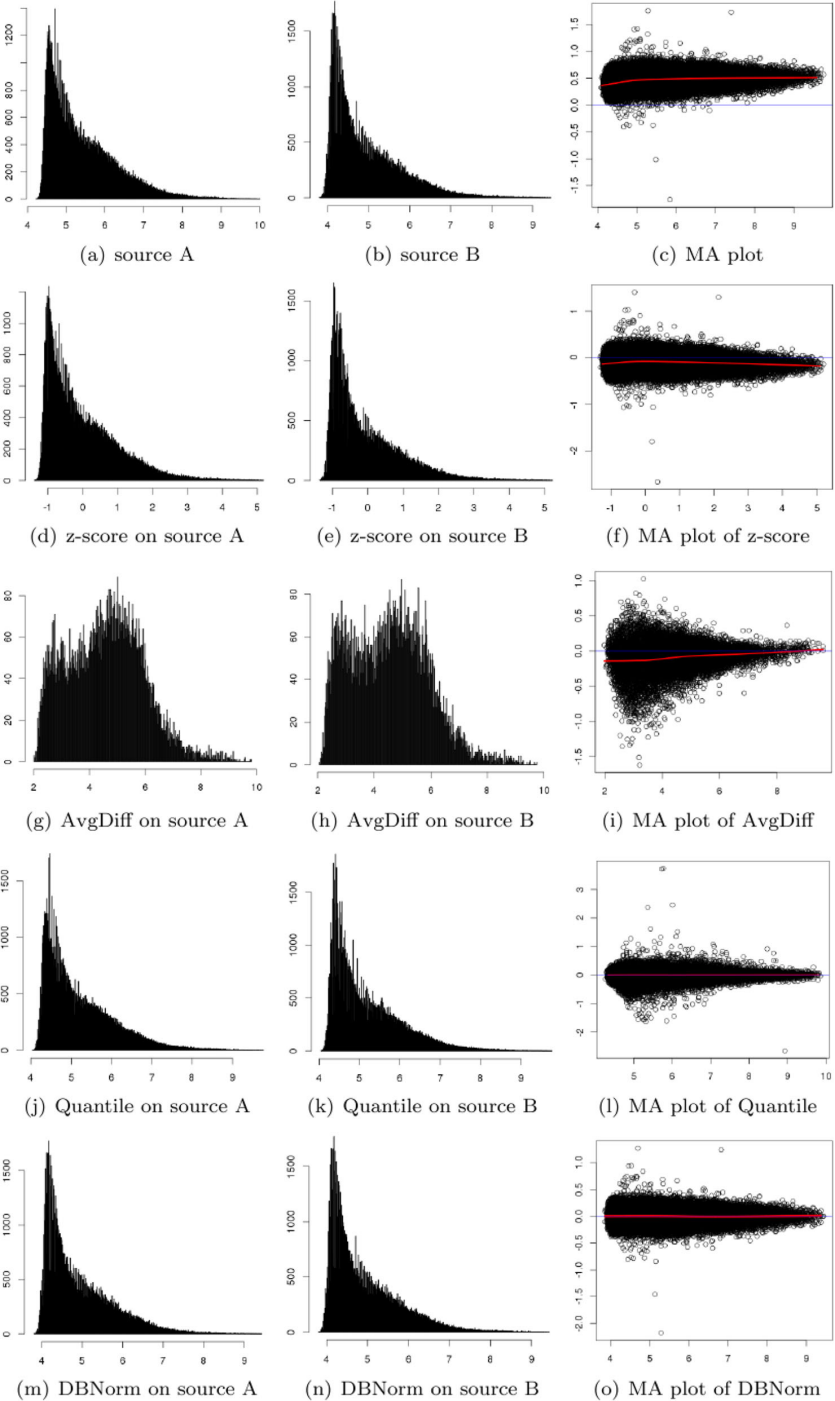
Visualization and comparison of normalization performance on Dilution/mixture dataset

For this experiment, we generate a dataset from both source A and source B. Specifically, we choose 201,800 Affymetrix microarray features. The results of normalization are illustrated in Fig. 4.

The dilution effect is clear in Fig. 4(c) where the overall ratio M is at 0.5. The red line is the row median of the standard Dilution/mixture dataset, while the blue line is a base. The effect of good normalization will bring the red line close to the blue line where M = 0. Furthermore, Figs. 4(a) and (b) show that the data from the two sources have different ranges and that their distributions have a slight difference.

Z-score normalization narrows the gap between the red and blue lines as illustrated in Fig. 4(f). However, the samples are widely spread at both sides. From Fig. 4(d) and (e), z-score normalization maps the data into the same range and keeps the distributions unchanged. This is the main reason why data samples are widely spread along the row median.

AvgDiff works better than z-score. From MA plot (Fig. 4(i)), we can see that AvgDiff minimizes red and blue lines in the initial part but the gap between them increases sharply. Fig. 4(g) and (h) are the distributions after AvgDiff normalization; the data are more evenly distributed compared with Figs. 4(a) and (b). This is why samples stay close in the MA plot. However, the distributions after AvgDiff normalization are still different, resulting in the gap between red and blue lines increasing.

Intuitively, both Quantile and DBNorm normalization can minimize the gap between the red and blue lines (Figs. 4(l) and (o)) in the MA plot. DBNorm works better than Quantile normalization. First, DBNorm achieves better values in Median and Interquartile Range (IQR), 0 and 0.138 respectively. Secondly, DBNorm compress Affymetrix microarray features into a smaller range, from −2.216 to 1.275.

The distributions of normalized data from DBNorm are more similar than those from Quantile normalization. The KL divergence of the distributions normalized by DBNorm is 2.283 × 10^−6^, while the divergence of the distributions normalized by Quantile normalization is 5.788 × 10^−5^.

#### Spike-in dataset

**Fig. 5:**
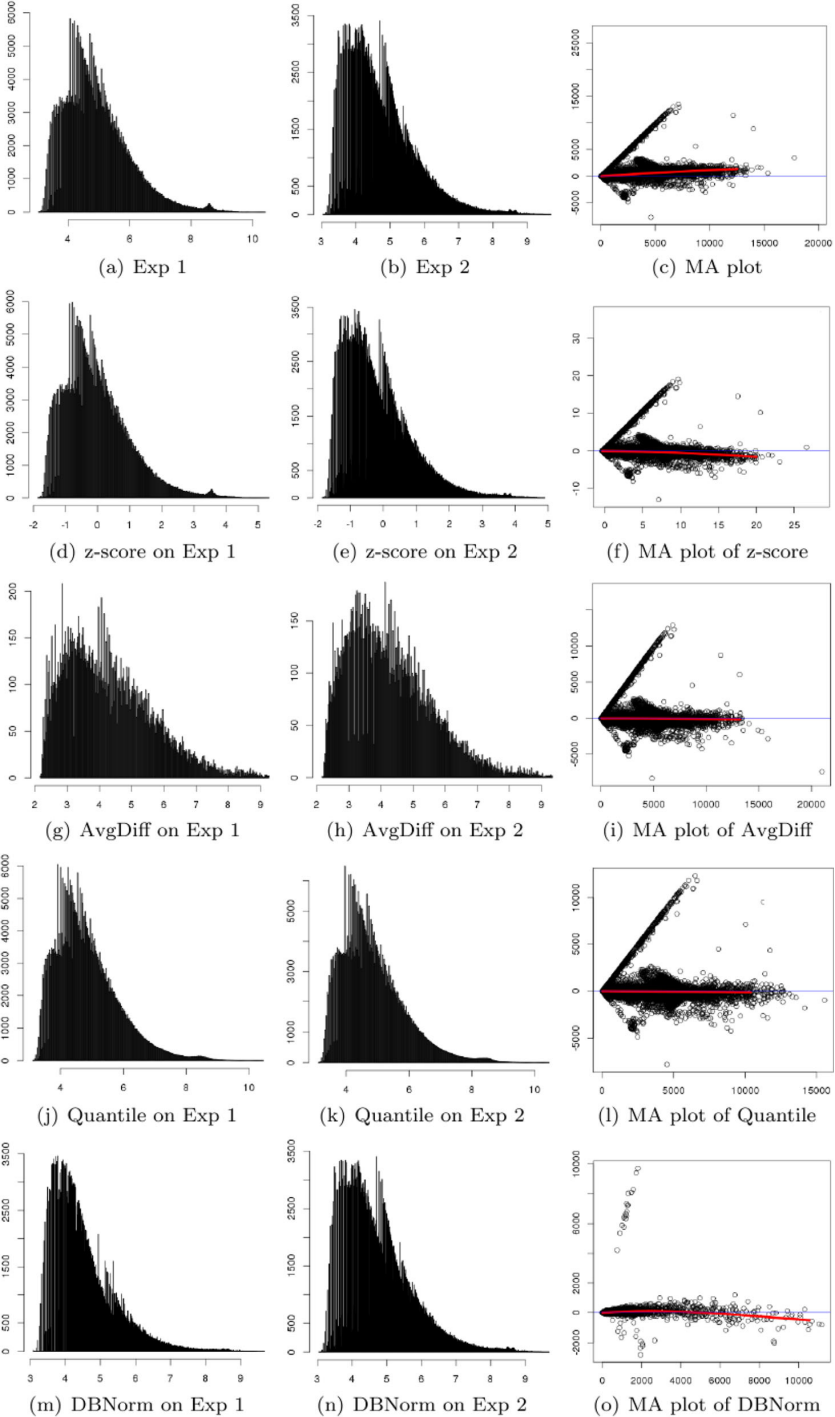
Visualization and comparison of normalization performance on Spike-in dataset

We further compare the performance of normalization on a dataset with 5184 common Affymetrix microarray features from platforms HGU133 and HGU95 to determine the ability of DBNorm on an atypical dataset that represents an extreme example of data distributions. The results of normalization are illustrated in Fig. 5.

From Fig. 5(c), we can see that the microarray data from these two selected experiments with the Spike-in dataset show clear artefacts from the spiked in genes, despite the data being generally well normalised based on the fact that the data sits along the blue axis. Also, Figs. 5(a) and (b) show that the data from these two experiments have different ranges and distributions.

During normalization by Z-score, AvgDiff and Quantile, the spiked in gene populations are resistant to their normalization effect as shown in Figs. 5(f), (i) and (l). DBNorm has the strongest effect among the four normalization methods, showing a powerful influence on disproportionate and extremely skewed data. In this extreme case, the power of the normalisation is evident. The spiked in peak is still seen in Fig. 5(n) despite a balancing of the overall distribution (see Fig. 5(m)) and the high level of compression observed in the MA plot (Fig. 5(o)). This level of change is not seen in the other examples. The normalized data sources show very similar distributions (Fig. 5(m) and (n)) leading to a good result in the MA plot in Fig. 5(o). The red and blue lines are very close. DBNorm compresses data into a very narrow range so that dots are overlapped dramatically.

#### Acute lymphoblastic leukemia (ALL) dataset

**Fig. 6:**
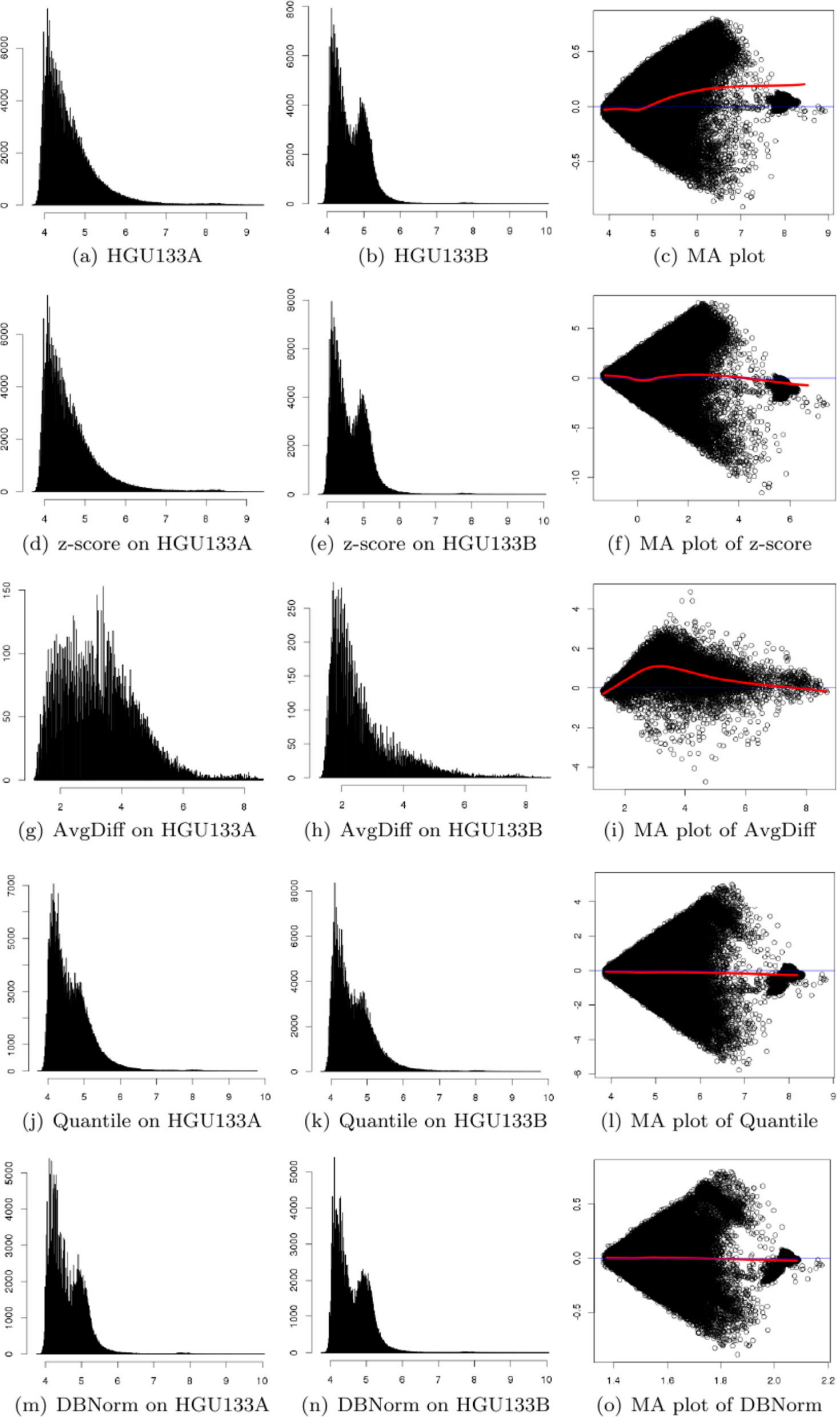
Visualization and comparison of normalization performance on the public ALL dataset

Finally, we compare the performance of normalization on a public MLL dataset generated from Affymetrix platforms HG-U133A and HG-U133B. The results of normalization are illustrated in Fig. 6. With this artificially configured data we expect a widely dispersed distribution of M values, which is what we see in Fig. 6(c). It also shows a shifting M ratio relative to spot intensity. We expect good normalization to remove this skew whilst maintaining the overall widely dispersed distribution. Z-score (Fig. 6(f)) and AvgDiff (Fig. 6(i)) normalization failed to do this. Quantile normalization and DBNorm (Figs. 6(l) and (o)) achieve better results because they focus on distributions. Quantile normalized data shows similar distributions in Fig. 6(j) and (k) but a wider dispersion in ratio. DBNorm is closer to the original with regards to the range dispersal (Fig. 6(m) and (n)).

## Conclusion

Microarray data from heterogeneous sources is common, but has been underexploited because of the difficulty of matching data from different platforms at different times. Normalization is the key to data integration and enables consistent downstream analysis. DBNorm outperforms the other three methods investigated, based on statistical properties, KL divergence, and classification.

## Additional files

Additional file 1: DBNorm user manual. A formal R package user manual which describes all functions contained in DBNorm package and examples. (PDF 104 kb)

Additional file 2: DBNorm testing document. Results of how we test DBNorm package with built-in datasets. (DOCX 575 kb)

Additional file 3: DBNorm test script. Code of how we test DBNorm package. (TXT 2 kb)

Additional file 4: DBNorm installation. Describes how to install DBNorm via devtools in R. (TXT 4 kb)

## Abbreviations

ALL: Acute Lymphoblastic Leukaemia
AvgDiff: Average Difference
DBNorm: Distribution-based Normalization
DS1: Data source 1
DS2: Data source 2
KL divergence: Kullback–Leibler divergence
LOOCV: Leave-one-out-cross-validation
PCA: Principal Component Analysis
SVM: Support Vector Machine
TC: Total Count
TMM: Trimmed Mean of M values

## Footnotes

1
http://www.affymetrix.com

2
https://www.schn.health.nsw.gov.au

3
http://bioconductor.org/packages/2.0/data/experiment/html/affydata.html

4
http://bioconductor.org/packages/2.0/data/experiment/html/SpikeIn.html

5
http://master.bioconductor.org/packages/2.0/data/experiment/html/ALLMLL.html
