# Supplementary material for: DBNorm: normalizing high-density oligonucleotide microarray data based on distributions
Source: BMC Bioinformatics. 2017 Nov 29;18:527. doi: 10.1186/s12859-017-1912-5 (PMC5706403; doi:10.1186/s12859-017-1912-5)
Supplement: Supplementary file 1 — DBNorm user manual. A formal R package user manual which describes all functions contained in DBNorm package and examples. (PDF 104 kb) [file 12859_2017_1912_MOESM1_ESM.pdf]

R documentation

of all in 'man'

October 9, 2016

R topics documented:

conNormalizer 1
custFit 2
defineDist 3
disNormalizer 4
distrNormalizer 5
fourierFit 5
gaussianFit 6
genDistData 7
loadData 8
polyFit 9
visDistData 9
visFitting 10

Index **12**

`conNormalizer` *Normalizing a target data array to a basis array based on their distributions*

Description

Normalizing a target data array to a basis array based on their distributions

Usage

```
conNormalizer(tg, bs)
```

Arguments

`tg`	a target data array
`bs`	a basis data array

Details

The function maps a target data array to a basis array based on their distributions and the basis data array can be an arbitary data array or a standard distribution such as normal distribution.

Value

A normalized target data array with the same distribution with the basis data array

Author(s)

Qinxue Meng, Paul Kennedy

Examples

```
# Normalize DArray1 to DArray3
# load build-in data arrays
data(DArray1)
data(DArray3)

# Capturing distribution information
DBdata1 <- genDistData(DArray1, 500)
DBdata3 <- genDistData(DArray3, 500)

# Using Gaussian function to fit DBdata3
DBdata3 <- gaussianFit(DBdata3)

# Normalize DBdata1 to the Gaussian fitting function of DBdata3
DArray1 = conNormalizer(DArray1, DArray3)
DA1toDA3DBdata <- genDistData(DA1toDA3, 500)
visDistData(DA1toDA3DBdata, "P", "DA1toDA3", "Range", "Probability")
```

`custFit` *fitting a distribution by a customised curve function*

Description

fitting a distribution by a customised curve function

Usage

```
custFit(DBdata, formula)
```

Arguments

`DBdata`	input distribution dataset
`formula`	a customised curve function

Details

The function fits distributions by a customised curve fitting and returns a customised curve fitting function.

Value

a customised curve fitting function

Author(s)

Qinxue Meng, Paul Kennedy

See Also

`lm`

Examples

```
# Calculating the customised curve fitting function of DArray1's distribution
DBdata1 = custFit(DBdata1)
```

`defineDist` *Generating distribution data based on predefined distribution*

Description

Generating distribution data based on predefined distribution

Usage

```
defineDist(dist)
```

Arguments

`dist`	a predefined distribution
`min`	the lower bound of data range and default value is 0
`max`	the upper bound of data range and default value is 1

Details

This function generates distribution data based on predefined distribution. The purpose of this function is to enable to normalize arbitrary distributions into a standard distribution.

Value

a distribution dataset of the input predefined distribution

Author(s)

Qinxue Meng, Paul Kennedy

See Also

`list()`

Examples

```
# generate distribution data of a normal distribution
DArray5 <- defineDist(Norm(mean=0, sd=1))
```

`disNormalizer` *Normalizing a target data array to a basis array based on element positions*

Description

Normalizing a target data array to a basis array based on element positions

Usage

```
disNormalizer(tg, bs)
```

Arguments

`tg`	a target data array
`bs`	a basis data array

Details

The function normalize target data array to a basis array based on element positions. This method does not need to do fitting before normalization and works for discrete values as well.

Value

A normalized target data array with the same distribution with the basis data array

Author(s)

Qinxue Meng, Paul Kennedy

Examples

```
# Calculating the polynomial curve fitting function of DArray1's distribution
DArray1 = disNormalizer(DArray1, DArray3)
```

distrNormalizer — *Normalizing a target data array to a standard distribution*

Description

Normalizing a target data array to a standard distribution

Usage

```
distrNormalizer(tg, bs)
```

Arguments

`tg`	a target data array
`bs`	a standard distribution created by defineDist(dist)

Details

The function normalize target data array to a standard distribution.

Value

A normalized target data array with the same distribution with the standard distribution

Author(s)

Qinxue Meng, Paul Kennedy

Examples

```
# Normalize a given data array into a normal distribution
loadData(0)
DBdata1 <- genDistData(DArray1, 500)
DBdata5 <- defineDist(Norm(mean=0, sd=1))
DA1toDA5 <- distrNormalizer(DBdata1, DBdata5)
DA1toDA5DBdata <- genDistData(DA1toDA5, 500)
visDistData(DA1toDA5DBdata, "P", "DA1toDA5", "Range", "Probability")
```

fourierFit — *fitting a distribution by fourier curve fitting*

Description

fitting a distribution by fourier curve fitting

Usage

```
fourierFit(DBdata, n)
```

Arguments

`DBdata`	input distribution dataset
`n`	the degree of the fourier fitting function

Details

The function fits distributions by fourier curve fitting and returns a fourier curve fitting function.

Value

a fourier curve fitting function

Author(s)

Qinxue Meng, Paul Kennedy

See Also

`lm`

Examples

```
# Calculating the fourier curve fitting function of DArray1's distribution
DBdata1 = fourierFit(DBdata1, 3)
```

`gaussianFit` *fitting a distribution by gaussian curve fitting*

Description

fitting a distribution by gaussian curve fitting

Usage

```
gaussianFit(DBdata)
```

Arguments

`DBdata`	input distribution dataset

Details

The function fits distributions by gaussian curve fitting and returns a gaussian curve fitting function.

Value

a gaussian curve fitting function

Author(s)

Qinxue Meng, Paul Kennedy

See Also

`optim`

Examples

```
# Calculating the gaussian curve fitting function of DArray1's distribution
DBdata1 = gaussianFit(DBdata1)
```

`genDistData` *Generating distribution dataset based on input data arrays.*

Description

Generating distribution dataset based on input data arrays.

Usage

```
genDistData(data, nbin)
```

Arguments

`data`	input data array
`nbin`	number of bins

Details

This function generates distribution dataset based on input data arrays for downstream analysis.

Value

a distribution dataset of a given input data array

Author(s)

Qinxue Meng, Paul Kennedy

See Also

`list()`

Examples

```
# load DArray1
DData1 <- genDistData(DArray1, 500)
```

`loadData` *Loading build-in datasets*

Description

This function loads build-in data array for examples

Usage

```
loadData(n)
```

Arguments

`n`	n-th data array to load; if n = 1, DArray1 is loaded; if n = 2, DArray2 is loaded; if n = 3, DArray3 is loaded; if n = 4, DArray4 is loaded; if n is not 1, 2, 3, 4, all four data arrays are loaded.

Details

This function loads example data arrays for user to test

Value

None

Author(s)

Qinxue Meng

See Also

`data()`

Examples

```
# load DArray1
loadData(1)
# load all data arrays
loadData(5)
```

`polyFit` *fitting a distribution by polynomial curve fitting*

Description

fitting a distribution by polynomial curve fitting

Usage

```
polyFit(DBdata, n)
```

Arguments

`DBdata`	input distribution dataset
`n`	the degree of polynomial functions

Details

The function fits distributions by polynomial curve fitting and returns a polynomial curve fitting function.

Value

a polynomial curve fitting function

Author(s)

Qinxue Meng, Paul Kennedy

See Also

`lm`

Examples

```
# Calculating the polynomial curve fitting function of DArray1's distribution
DBdata1 = polyFit(DBdata1, 3)
```

`visDistData` *Visualising distribution dataset*

Description

Visualising distribution dataset

Usage

```
visDistData(DBdata, type, t, xl, yl)
```

Arguments

`DBdata`	a distribution dataset
`type`	plot by frequence / probability
`t`	title of plot
`xl`	description of x-asis
`yl`	description of y-asis

Details

This function generates distribution data based on predefined distribution. The purpose of this function is to enable to normalize arbitrary distributions into a standard distribution.

Author(s)

Qinxue Meng, Paul Kennedy

Examples

```
# visualising a distribution data
DBdata1 <- genDistData(DArray1, 500)
visDistData(DBdata1, "F", "DArray1", "Range", "Frequence")
visDistData(DBdata1, "P", "DArray1", "Range", "Probability")
```

`visFitting` *Visualising fitting results on the input distribution*

Description

Visualising fitting results on the input distribution

Usage

```
visFitting(DBdata, t, xl, yl)
```

Arguments

`DBdata`	a distribution dataset
`t`	title of plot
`xl`	description of x-asis
`yl`	description of y-asis

Details

The function visualizes data distribution and corresponding fitting function so as to provide an intutive way to evaluate the performance of fitting function.

Author(s)

Qinxue Meng, Paul Kennedy

Examples

```
# visualising fitting results on DArray1's distribution
visFitting(DBdata1, "DArray1", "Range", "Probability")
```

Index

conNormalizer, 1
custFit, 2

defineDist, 3
disNormalizer, 4
distrNormalizer, 5

fourierFit, 5

gaussianFit, 6
genDistData, 7

loadData, 8

polyFit, 9

visDistData, 9
visFitting, 10
