# Supplementary material for: DBNorm: normalizing high-density oligonucleotide microarray data based on distributions
Source: BMC Bioinformatics. 2017 Nov 29;18:527. doi: 10.1186/s12859-017-1912-5 (PMC5706403; doi:10.1186/s12859-017-1912-5)
Supplement: Supplementary file 2 — DBNorm testing document. Results of how we test DBNorm package with built-in datasets. (DOCX 575 kb) [file 12859_2017_1912_MOESM2_ESM.docx]

DBNorm

# Build-in dataset

DBNorm library provides four build-in data arrays for testing and they are DArray1 (22,277), DArray2 (22,277), DArray3 (54,675) and DArray4 (33,297). These four data arrays can be loaded via function data() or loadData().

| # load build-in data arrays by data()  data(DArray1)  data(DArray2)  data(DArray3)  data(DArray4)  # or load build-in data arrays by loadData()  loadData(0) |
| --- |

# Define standard distribution

The library allows user to define standard distributions for normalizing arbitrary distributions into standard ones.

| # define a normal distribution  DBdata5 <- defineDist(Norm(mean=0, sd=1), -5, 5) |
| --- |

# Visualizing distributions

The distributions of data arrays can be visualized by frequency or probability.

| **Frequency distribution** | **Probability distribution** |
| --- | --- |
| 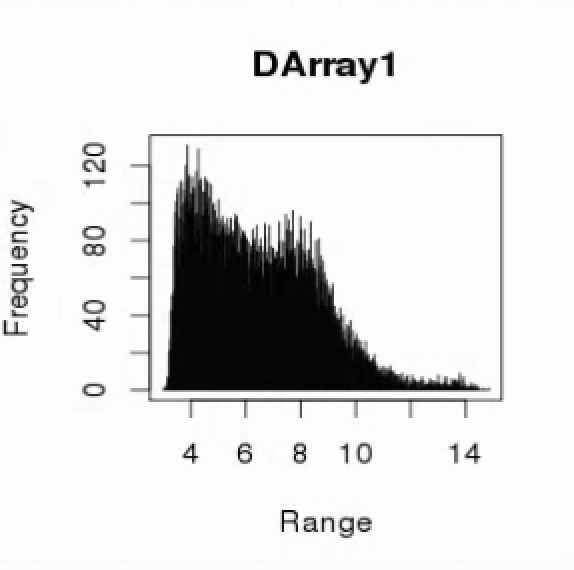 | 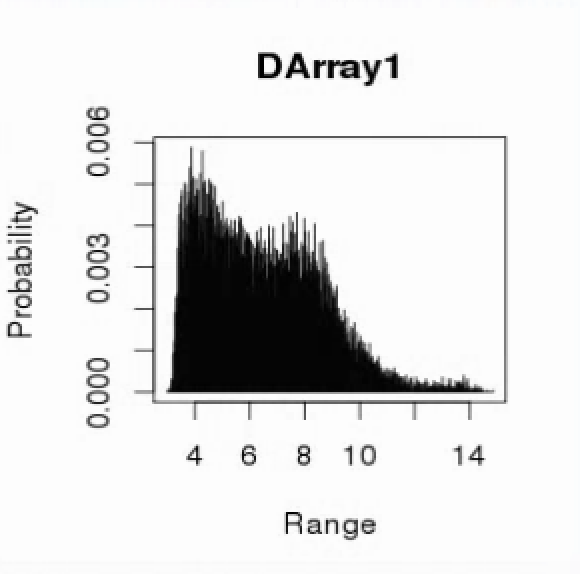 |
| # Frequency distribution of DArray1  visDistData(DBdata1, "F", "DArray1", "Range", "Frequency")  # Probability distribution of DArray1  visDistData(DBdata1, "P", "DArray1", "Range", "Probability") | |

# Fitting distributions and visualization

The library provides several ways of fitting distributions, visualizing fitting results on distributions and generate fitting formula for normalization.

## Polynomial fitting

Users can define the degree of polynomial equations to fit. For example, n-degree polynomial equation is defined as

$$y=a_{0}+a_{1}x+a_{2}x^{2}+\ldots+a_{n}x^{n}$$

| **A 9-degree polynomial fitting** | **Formula** |
| --- | --- |
| 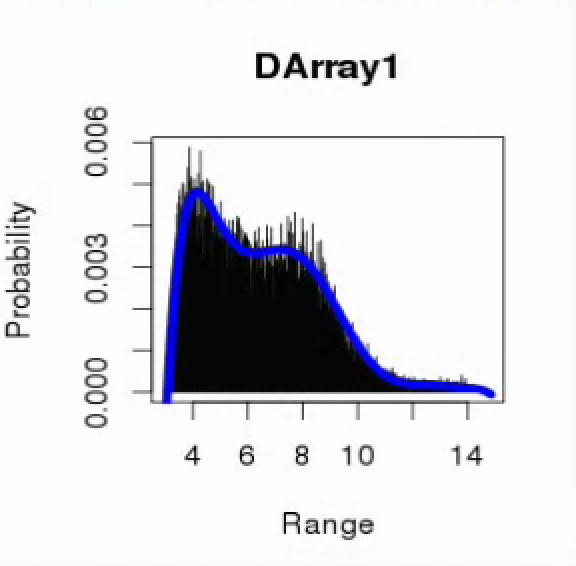 | y = (-0.000620900046122378)*x^9+(-0.00124834884502108)*x^8+(0.00662249052719529)*x^7+(-0.0084800462241037)*x^6+(0.00310757525823036)*x^5+(-0.00428172357339282)*x^4+(0.0130352656779109)*x^3+(-0.00443148578371705)*x^2+(-0.0320215363793821)*x^1+(0.00199999999999999) |

| # Fitting DArray’s distribution by a 9-degree polynomial equation  DBdata1 <- polyFit(DBdata1, 9)  # Visualize fitting results  visFitting(DBdata1, "DArray1", "Range", "Probability")  # Fitting equation  DBdata1$equ |
| --- |

## Fourier fitting

Users can define the degree of Fourier equations to fit. For example, n-degree Fourier equation is defined as

$$y=a_{0}+a_{1}\cos\left( w*x \right)+b_{1}\sin\left( w*x \right)+\ldots+a_{n}\cos\left( n*w*x \right)+b_{n}\sin\left( n*w*x \right)$$

| **A 3-degree Fourier fitting** | **Formula** |
| --- | --- |
| 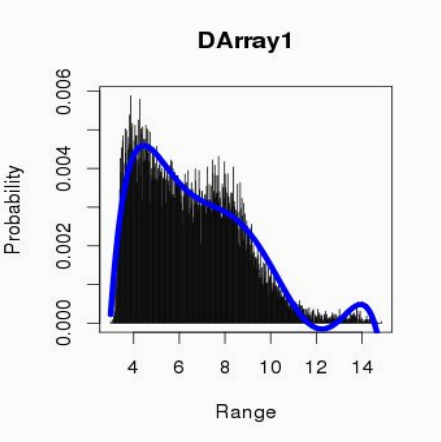 | y = -190.023896634087 + 244.79420434595*cos(0.0600018263994387*1*x) + 148.946640309215*sin(0.0600018263994387*1*x) + 148.946640309215*cos(0.0600018263994387*2*x) + -53.5495838217859*sin(0.0600018263994387*2*x) + -53.5495838217859*cos(0.0600018263994387*3*x) + -103.409272641898*sin(0.0600018263994387*3*x) |

| # Fitting DArray’s distribution by a 3-degree Fourier equation  DBdata1 <- fourierFit(DBdata1, 3)  # Visualize fitting results  visFitting(DBdata1, "DArray1", "Range", "Probability")  # Fitting equation  DBdata1$equ |
| --- |

## Gaussian fitting

Users can use Gaussian equations to fit. For example, the Gaussian equation in the library is defined as

$$y=a*e^{\frac{{(x-\mu)}^{2}}{\sigma^{2}}}$$

| **Gaussian fitting** | **Formula** |
| --- | --- |
| 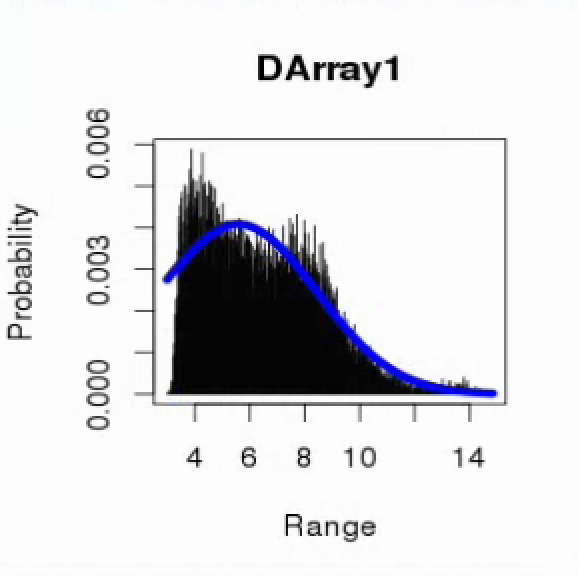 | y = (0.0293110169514227)*exp^(-(2.86021073423377)*(x-5.54082132297444)^2) |

| # Fitting DArray’s distribution by Gaussian equation  DBdata1 <- gaussianFit(DBdata1)  # Visualize fitting results  visFitting(DBdata1, "DArray1", "Range", "Probability")  # Fitting equation  DBdata1$equ |
| --- |

## Customized fitting

Users can define an equation to fit. For example, we define an equation as

$$y=a*x+b*\cos\left( x \right)+c$$

| **Gaussian fitting** | **Formula** |
| --- | --- |
| 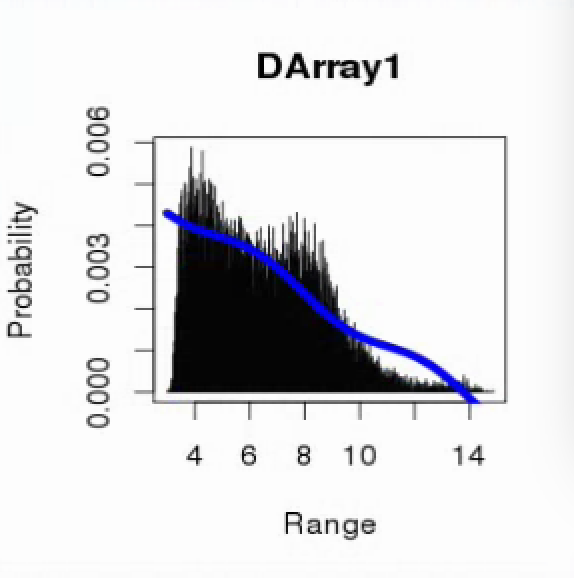 | y = (0.0057963567671893) + (-0.000426884293350865)*x + (0.000221349815448716)*cos(x) |

| # Fitting DArray’s distribution by a customized equation  DBdata1 = custFit(DBdata1, "y ~ x + cos(x)")  # Visualize fitting results  visFitting(DBdata1, "DArray1", "Range", "Probability")  # Fitting equation  DBdata1$equ |
| --- |

# Normalization

The library offers the function to normalize an arbitrary data array to another one and the performance of normalization is determined by the fitting functions.

## conNormalizer: normalizing between two arbitrary distributions by conNormalizer

### Good fitting

| **Before normalization** | |
| --- | --- |
| 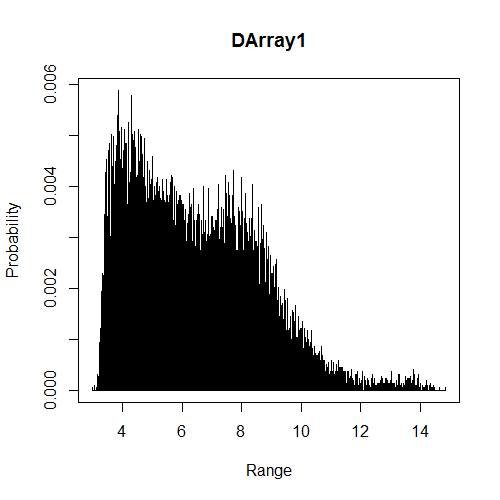 | 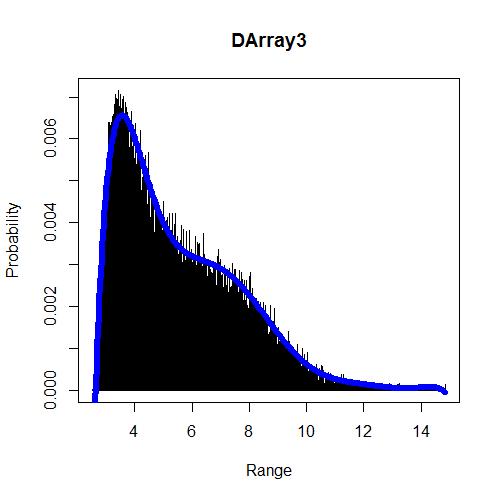 |
| **After normalization** | |
| 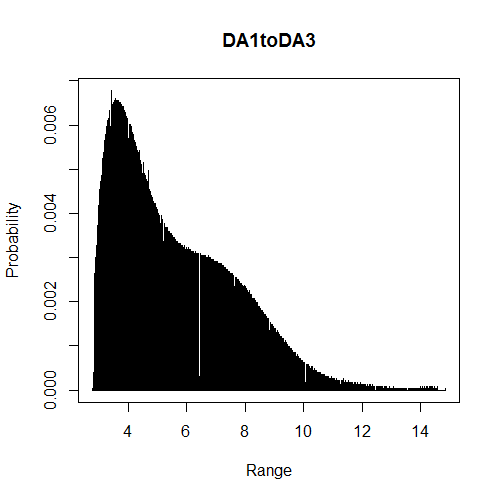 | 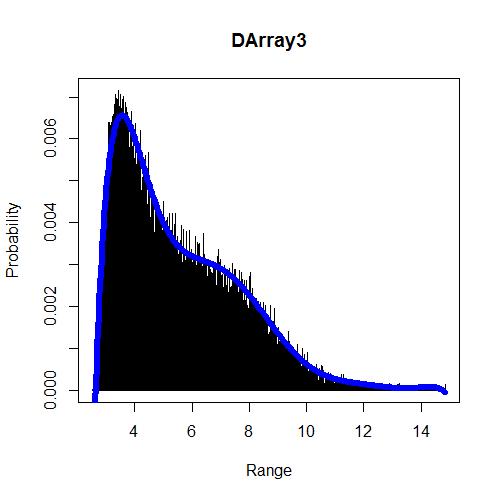 |

| # load build-in data arrays  data(DArray1)  data(DArray3)  # Capturing distribution information  DBdata1 <- genDistData(DArray1, 500)  DBdata3 <- genDistData(DArray3, 500)  # Using Gaussian function to fit DBdata3  DBdata3 <- polyFit(DBdata3, 9)  # Normalize DBdata1 to the Gaussian fitting function of DBdata3  DArray1 = conNormalizer(DArray1, DArray3)  DA1toDA3DBdata <- genDistData(DA1toDA3, 500)  visDistData(DA1toDA3DBdata, "P", "DA1toDA3", "Range", "Probability") |
| --- |

### Bad fitting

| **Before normalization** | |
| --- | --- |
| 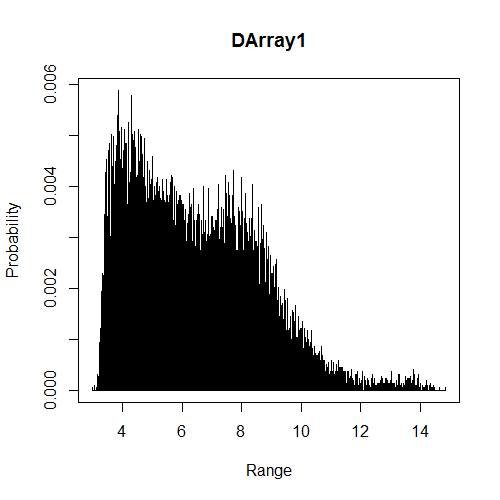 | 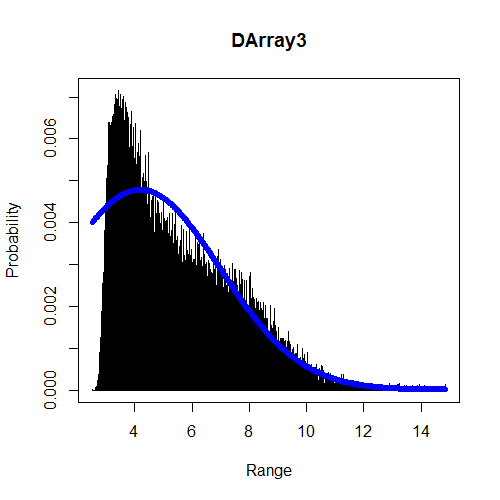 |
| **After normalization** | |
| 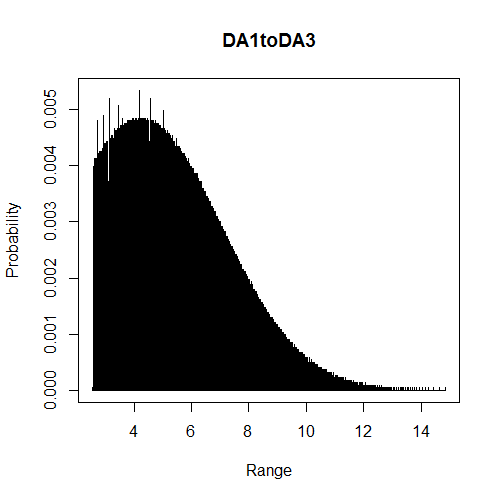 | 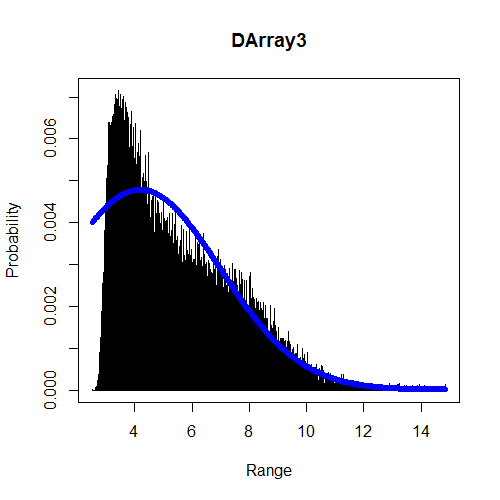 |

| # load build-in data arrays  data(DArray1)  data(DArray3)  # Capturing distribution information  DBdata1 <- genDistData(DArray1, 500)  DBdata3 <- genDistData(DArray3, 500)  # Using Gaussian function to fit DBdata3  DBdata3 <- gaussianFit(DBdata3)  # Normalize DBdata1 to the Gaussian fitting function of DBdata3  DArray1 = conNormalizer(DArray1, DArray3)  DA1toDA3DBdata <- genDistData(DA1toDA3, 500)  visDistData(DA1toDA3DBdata, "P", "DA1toDA3", "Range", "Probability") |
| --- |

The result of normalization is determined by how good we can fit the data distribution. A good fitting function can rescale the data arrays into the same distributions. If it is hard to achieve a good fitting functions, it is recommended to use disNormalizer() and distrNormalizer().

## disNormalizer: normalizing between two arbitrary distributions

| **Before normalization** | |
| --- | --- |
| 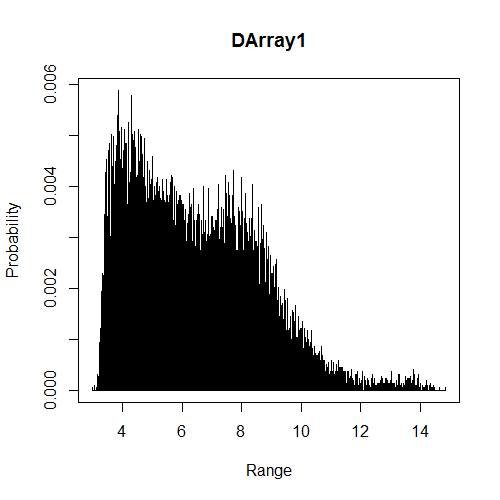 | 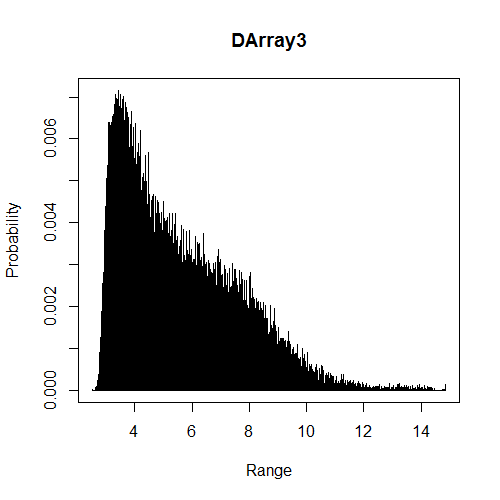 |
| **After normalization** | |
| 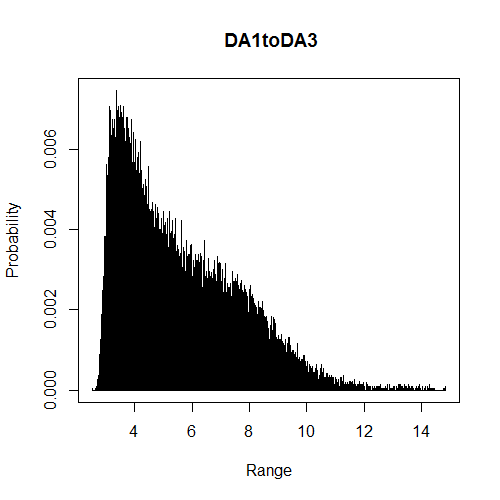 | 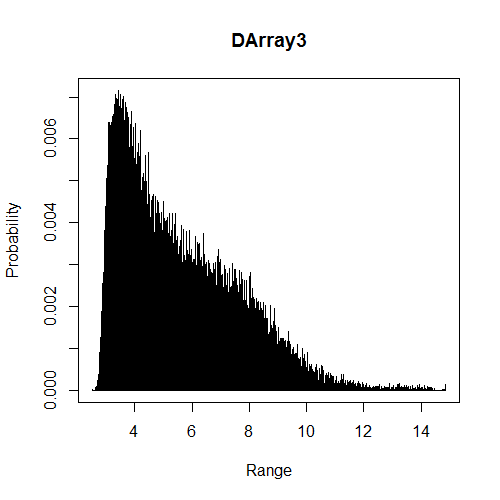 |

| # load build-in data arrays  data(DArray1)  data(DArray3)  # Capturing distribution information  DBdata1 <- genDistData(DArray1, 500)  DBdata3 <- genDistData(DArray3, 500)  # Discrete normalization  DA1toDA3 = disNormalizer(DBdata1$data, DBdata3$data)  DA1toDA3DBdata <- genDistData(DA1toDA3, 500)  visDistData(DA1toDA3DBdata, "P", " DA1toDA3", "Range", "Probability") |
| --- |

## distrNormalizer: normalizing an arbitrary distribution into a standard one

| **Before normalization** | |
| --- | --- |
| 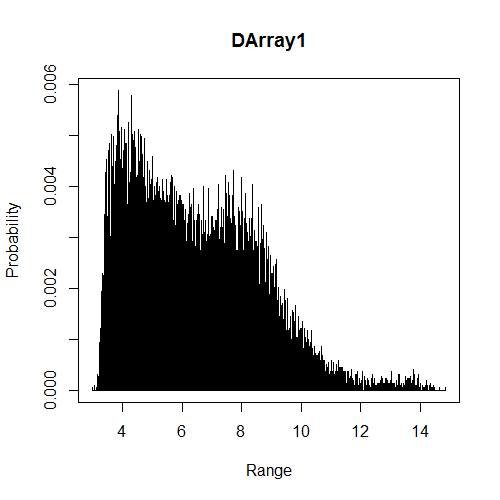 | 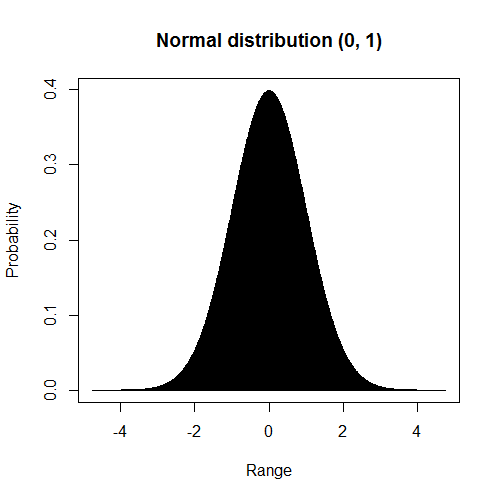 |
| **After normalization** | |
| 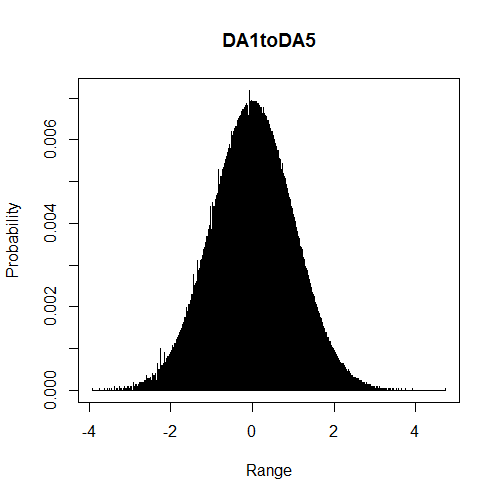 | 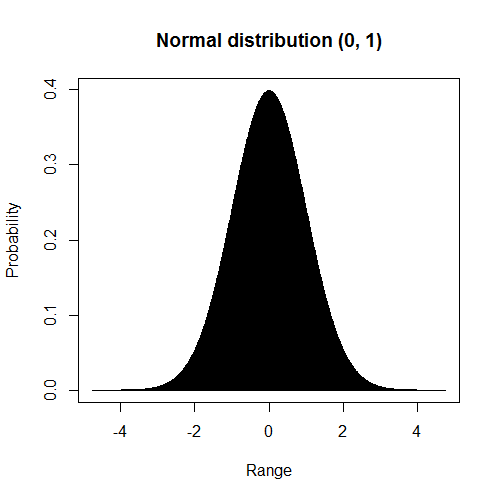 |

| # load build-in data arrays  data(DArray1)  DBdata5 <- defineDist(Norm(mean=0, sd=1))  # Capturing distribution information  DBdata1 <- genDistData(DArray1, 500)  # Distribution Normalization  DA1toDA5 = distrNormalizer(DBdata1, DBdata5)  DA1toDA5DBdata <- genDistData(DA1toDA5, 500)  visDistData(DA1toDA5DBdata, "P", "DA1toDA5", "Range", "Probability") |
| --- |
